# Interpretable Multi-Scale Deep Learning for RNA Methylation Analysis across Multiple Species

**DOI:** 10.3390/ijms25052869

**Published:** 2024-03-01

**Authors:** Rulan Wang, Chia-Ru Chung, Tzong-Yi Lee

**Affiliations:** 1 Shenzhen Institute of Advanced Technology, Chinese Academy of Sciences, Shenzhen 518055, China; rl.wang1@siat.ac.cn; 2 Department of Computer Science and Information Engineering, National Central University, Taoyuan 320317, Taiwan; 3 Institute of Bioinformatics and Systems Biology, National Yang Ming Chiao Tung University, Hsinchu 30010, Taiwan

**Keywords:** RNA modification, multi-scale biological information analysis, language-based deep learning model, interpretable prediction

## Abstract

RNA modification plays a crucial role in cellular regulation. However, traditional high-throughput sequencing methods for elucidating their functional mechanisms are time-consuming and labor-intensive, despite extensive research. Moreover, existing methods often limit their focus to specific species, neglecting the simultaneous exploration of RNA modifications across diverse species. Therefore, a versatile computational approach is necessary for interpretable analysis of RNA modifications across species. A multi-scale biological language-based deep learning model is proposed for interpretable, sequential-level prediction of diverse RNA modifications. Benchmark comparisons across species demonstrate the model’s superiority in predicting various RNA methylation types over current state-of-the-art methods. The cross-species validation and attention weight visualization also highlight the model’s capability to capture sequential and functional semantics from genomic backgrounds. Our analysis of RNA modifications helps us find the potential existence of “biological grammars” in each modification type, which could be effective for mapping methylation-related sequential patterns and understanding the underlying biological mechanisms of RNA modifications.

## 1. Introduction

RNA modification is a crucial process that plays a vital role in various cellular processes, including gene silencing, protection against spurious repetitive element activity, genomic stability during mitosis, and parent-of-origin imprinting [1]. Post-transcriptional modifications of RNA molecules are diverse and can involve N1-methyladenosine (m1A, catalyzed by methyl-transferases, which add a methyl group to the nitrogen at the first position of the adenosine base [2]), N6-methyladenosine (m6A, involving methylation modification of the nitrogen at the sixth position of the adenosine base [3,4]), and pseudouridine (Ψ or pseU, produced by the isomerization of uridine), named according to the type of nucleotide, the type of molecule added, and the position of modification within the nucleotide [5]. Recent studies show that RNA post-transcriptional modifications play crucial roles in many physiological behaviors, including the regulation and development of various diseases such as cancer, psychiatric disorders, and metabolic disease [2,6,7], for example, m1A and m6A modifications can influence the structure and function of both transfer RNA (tRNA) and ribosomal RNA (rRNA) and play a broad and crucial role in almost all aspects of physiological behavior [3,4,8,9]. Pseudouridine has been shown to play vital roles in molecular mechanisms, such as stabilizing RNA structure, facilitating RNA-protein or RNA-RNA interactions, regulating the entry site binding process, and influence the metabolism of RNA structure [10,11,12,13,14,15].

The development of uncovering mechanisms for RNA modifications has undergone a long journey. Benefiting from the rapid development of next-generation sequencing technology, biologists are now able to experimentally identify various types of RNA modifications, due to recent advances in genomics and molecular biology. Consequently, more than 160 modifications have been identified within RNAs, and these are deposited and annotated in public databases [16]. The identification of experimentally validated RNA modifications has proven useful in revealing their important patterns and novel functions. Due to the high consumption of time and labor in experimental verification techniques, computational methods have become increasingly popular for predicting RNA modifications in recent years, with a range of techniques employed from conventional machine learning methods [17,18,19,20] to deep learning methods [21,22,23], depending on the dataset scale. Table 1 provides some examples of representative RNA modification predictors.

Although bioinformatics tools are effective, they face challenges due to the rapidly expanding volume of experimentally verified data, which outpaces the capabilities of traditional machine learning methods. In contrast, deep learning approaches, such as DeepPromise [21], have shown promise in identifying multiple RNA modification sites, such as m1A and m6A, within transcriptomes. However, predicting other types of modifications using these models remains a challenge. Furthermore, although deep learning methods are proficient in handling large datasets, they frequently struggle to identify the essential sequential patterns that are critical for comprehending RNA methylation mechanisms. This limitation impedes the interpretability of these models and our overall understanding of RNA methylation prediction. While some models, such as MultiRM [24], have attempted to improve interpretability, there are still concerns about their ability to distinguish the crucial information encoded in genomic sequences that support RNA methylation mechanisms.

**Figure 1:**
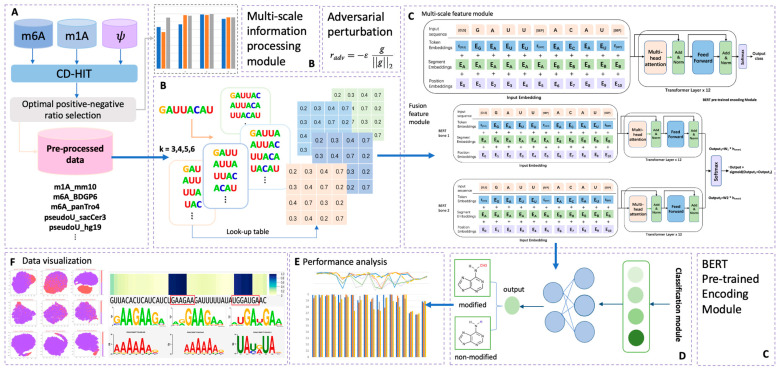
Overall framework of the predictors used in this research. Part (**A**) illustrates the data pre-processing phase; in this step, sample data of three different modifications were collected. After CD-HIT and positive–negative ratio adjustment pre-processing, eighteen totally processed datasets were collected for later prediction. Part (**B**) illustrates the multi-scale information processing module; four different choices of k-mer features were encoded in this step, with k ranging from three to six. Part (**C**) illustrates the overall structure of the BERT encoder module, which contains two main model types in this research: multi-scale feature module and fusion feature module. In this part, adversarial perturbation function was adopted for obtaining a robust model. Part (**D**) illustrates the output phase from Part (**C**), in which the BERT model’s output was classified into two types: modified (positive) and non-modified (negative). Performance analysis and data visualization were also carried out, as indicated in phases (**E**,**F**).

To address these limitations, we propose BERT-RNA, a model that utilizes pretraining on genomic sequences to understand biological context and identify unique methylation patterns across different scales. Additionally, we have incorporated adversarial training to enhance the model’s predictive accuracy and robustness. Our approach includes an interpretable analysis framework that focuses on local sequence features through attention mechanisms. This framework offers insights into the sequential regions significantly associated with specific modifications. The possibility of ‘biological language grammars’ that govern and influence biological functions is suggested. The methodology and results of this study are summarized in Figure 1, demonstrating the potential of BERT-RNA in enhancing our comprehension of RNA modifications.

**Table 1:** Summary of representative methylation predictors in published work.

Tool Name	Modification	Method	Publication Year	Feature Selection Method ^1^	Feature Encoding Scheme ^2^	Data Scale (Sample Number)	URL Accessibility	Window Size	Species	Reference
RAMPred	m1A	SVM	2016	None	NCP, ANF	6366 (*H. sapiens*), 1064 (*Mus musculus*), 483 (*S. cerevisiae*)	Accessible	41	*H. sapiens*, *M. musculus*, and *S. cerevisiae*	[17]
iRNA-3typeA	m1A and m6A	SVM	2018	None	NCP, ANF	6366 (*H. sapiens*; m1A), 1064 (*Mus musculus*; m1A), 1130 (*H. sapiens*; m6A), 725 (Mus musculus; m6A)	Accessible	41	*H. sapiens* and *Mus musculus*	[18]
M6APred-EL	m6A	Ensemble SVM	2018	None	position-specific information, physical-chemical properties, ring-function -hydrogen-chemical properties	1307	Inaccessible	51	*S. cerevisiae*	[19]
iRNA(m6A)- PseDNC	m6A	SVM	2018	None	PseDNC	1307	Inaccessible	51	*S.* *cerevisiae*	[25]
BERMP	m6A	BGRU	2018	None	ENAC, RNA word embedding	53,000 (mammalian full transcript mode) 44,853 (mammalian mature mRNA mode) 1100 (*S. cerevisiae*) 2100 (*A. thaliana*)	Inaccessible	251 (Mammalian) 51 (*S. cerevisiae*) and 101 (*A. thaliana*)	*H. sapiens*, *M. musculus*, *S. cerevisiae* and *A. thaliana*	[26]
M6AMRFS	m6A	XGBoost	2018	SFS	DNC, binary, Local position-specific dinucleotide frequency	1307 (*S. cerevisiae*), 1130 (*H. sapiens*), 725 (Mus musculus) and 1000 (*A. thaliana*)	-	51 (*S. cerevisiae*), 41 (*H. sapiens*), 41 (*Mus musculus*) and 25 (*A. thaliana*)	*H. sapiens*, *M. musculus*, *S. cerevisiae* and *A. thaliana*	[27]
RFAthM6A	m6A	RF	2018	None	PSNSP, PSDSP, KSNPF, k-mer	2518	Accessible	101	*A. thaliana*	[28]
DeepM6APred	m6A	SVM	2019	None	deep features and NPPS	1307	Inaccessible	51	*S.* *cerevisiae*	[29]
Gene2Vec	m6A	CNN	2019	None	One-hot, Neighboring methylation state, RNA word embedding, Gene2Vec	56,557	Inaccessible	1001	*H. sapiens* and *Mus musculus*	[22]
WHISTLE	m6A	SVM	2022	perturb method	NCP, GNF, Genome-derived features	20,516, 17,383	Accessible	-	*H. sapiens*	[30]
DeepPromise	m6A	CNN	2022	None	ENAC, one-hot and RNA word embedding	44,901, 11,656 and 5233	Accessible	1001	*H. sapiens* and *Mus musculus*	[21]
Adaptive-m6A	m6A	Adaptive learning network	2023	CHI2	NAC, DNC, TNC, BE, CKSNAP, ENAC, NCP and RNA word embedding	6728 (*D. melanogaster*), 43,025 (zebrafish), 2172 (*E. coli*), 44,445 (Mus musculus), 2614 (*S. cerevisiae*) 5033 (*A. thaliana*)	Accessible	21	*D. melanogaster*, zebrafish, *E. coli*, *Mus musculus*, *S. cerevisiae A. thaliana*	[31]
PPUS	Ψ	SVM	2015	Dynamic window size	One-hot	464 (yeast), 102 (*H. sapiens*)	Accessible	dynamically	Yeast and *H. sapiens*	[32]
iRNA-PseU	Ψ	SVM	2016	None	NCP, ND, PseKNC	314 (*H. sapiens*), 495 (*S. cerevisiae*) and 314 (Mus musculus)	Accessible	5, 10, 15, 20	*S. cerevisiae*, *H. sapiens* and *Mus musculus*	[33]
PseUI	Ψ	SVM	2018	SFS	NAC, DNC, PseDNC, PSNP, PSDP	314 (*H. sapiens*), 495 (*S. cerevisiae*) and 314 (Mus musculus)	Inaccessible	21, 31	*S. cerevisiae*, *H. sapiens* and *Mus musculus*	[34]
iPseU-CNN	Ψ	CNN	2019	None	One-hot	990 (*H. sapiens*), 628 (*S. cerevisiae*) and 944 (*M. musculus*)	-	15	*S. cerevisiae*, *H. sapiens* and *Mus musculus*	[35]
EnsemPseU	Ψ	Ensemble	2020	CHI2, mRMR, F-score	Kmer, one-hot, ENAC, NCP, ND	990 (*H. sapiens*), 628 (*S. cerevisiae*) and 944 (*M. musculus*)	Inaccessible	-	*S. cerevisiae*, *H. sapiens* and *Mus musculus*	[36]
PIANO	Ψ	SVM	2020	None	SCP, PSNP, Genome-derived features	3566 (*H. sapiens*)	Accessible	41	*H. sapiens*	[37]
PSI-MOUSE	Ψ	SVM	2020	None	NCP, Genome-derived features	628 (*S. cerevisiae*) and 944 (*M. musculu*s)	Accessible	-	*S. cerevisiae*, *Mus musculus*	[38]
BERT2OME	2′-O-methylation	BERT	2023	None	RNA word embedding	1089 (*H. sapiens*), 278 (*S. cerevisiae*) and 45 (*M. musculu*s)	Inaccessible	41	*S. cerevisiae*, *H. sapiens* and *Mus musculus*	[39]
MSCAN	m^6^A, m^1^A, m^5^C, m^5^U, m^6^Am, m^7^G, Ψ, I, Am, Cm, Gm, and Um	multi-scale self- and cross-attention mechanisms	2024	None	RNA word embedding	9115 (m1A), 47,208 (m6A), 6803 (m5C), 986 (m5U), 1339 (m6Am), 691 (m7G), 2273 (Ψ), 4547 (Am, Cm, Gm, and Um) and 5901 (I)	Accessible	21, 31 and 41	*H. sapiens*	[40]

^1^ Abbreviation in feature selection method: SFS, sequence forward search; CHI2: Chi-squared method; mRMR: minimal Redundancy Maximum Relevance. ^2^ Abbreviation in feature encoding scheme: PseDNC, pseudo dinucleotide composition; NCP, nucleotide chemical property; ANF, accumulated nucleotide frequency; DNC, dinucleotide composition; PSNP, position-specific nucleotide propensity; PSDP, position-specific dinucleotide propensity; NPPS, nucleotide pair position specificity; ENAC, enhanced nucleic acid composition; PSNSP, position-specific nucleotide sequence profile; PSDSP, position-specific dinucleotide sequence profile; KSNPF, K-spaced nucleotide pair frequencies. ND: nucleotide density.

## 2. Results

### 2.1. Elucidate Methylation Mechanisms Based on Multi-Scale Sequential Design

**Figure 2:**
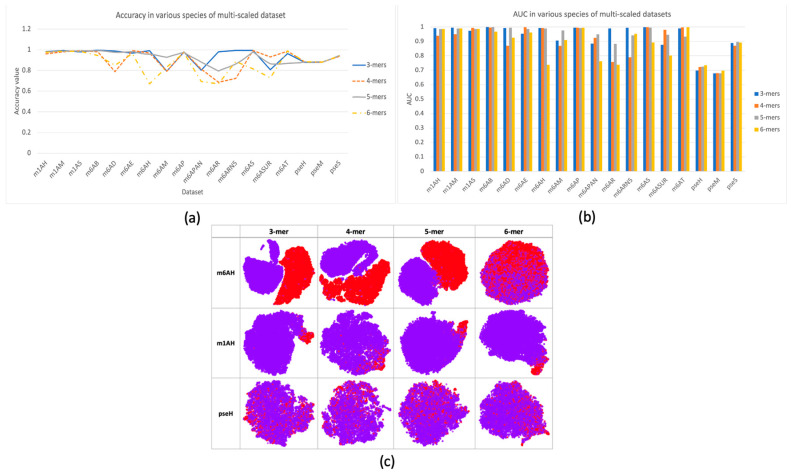
Performance and feature space distribution (with TSNE visualization) of multi-scale features in three different modifications of the *H. sapien* species: m6A, m1A, and Ψ. In (**a**), the criterion accuracy of multi-scaled features on various modifications is displayed. The criterion AUC is shown on the bar chart of (**b**). The feature space distribution visualization (**c**) shows negative (in purple color) and positive (in red color), which represent non-modified and true modified samples, respectively. The table-formed results for multi-scale features have been included in the supplementary file, Table S3.

In this research, a multi-scale information processing strategy was proposed to represent different “biological words” for feature representation. We compared different k-mers, with k ranging from 3 to 6. The comparison results were illustrated in Figure 2a,b with respect to criterion accuracy and AUC, respectively. It can be observed that different k-mers have their own advantages in different species of data, and no consistent good result was observed. Therefore, using single-scale sequential patterns may not sufficiently capture the inherent characteristics of methylations. This could be proved by the feature space distribution with TSNE visualization in Figure 2c, where it seems hard to find a specific k choice that provides the best separation between positive and negative samples in each dataset. In datasets m6AH and m1AH, there are noticeable differences between positive and negative samples, as evidenced by a larger gap between the positive and negative clusters. However, in the case of the Ψ modification dataset, the two clusters are often intermixed. On the other hand, in datasets with m1A modification, even with an uneven distribution of positive and negative samples, the clustering result is much better. Positive samples, represented by the red cluster, are prominently concentrated in one corner in most cases, with only minor mixtures.

**Figure 3:**
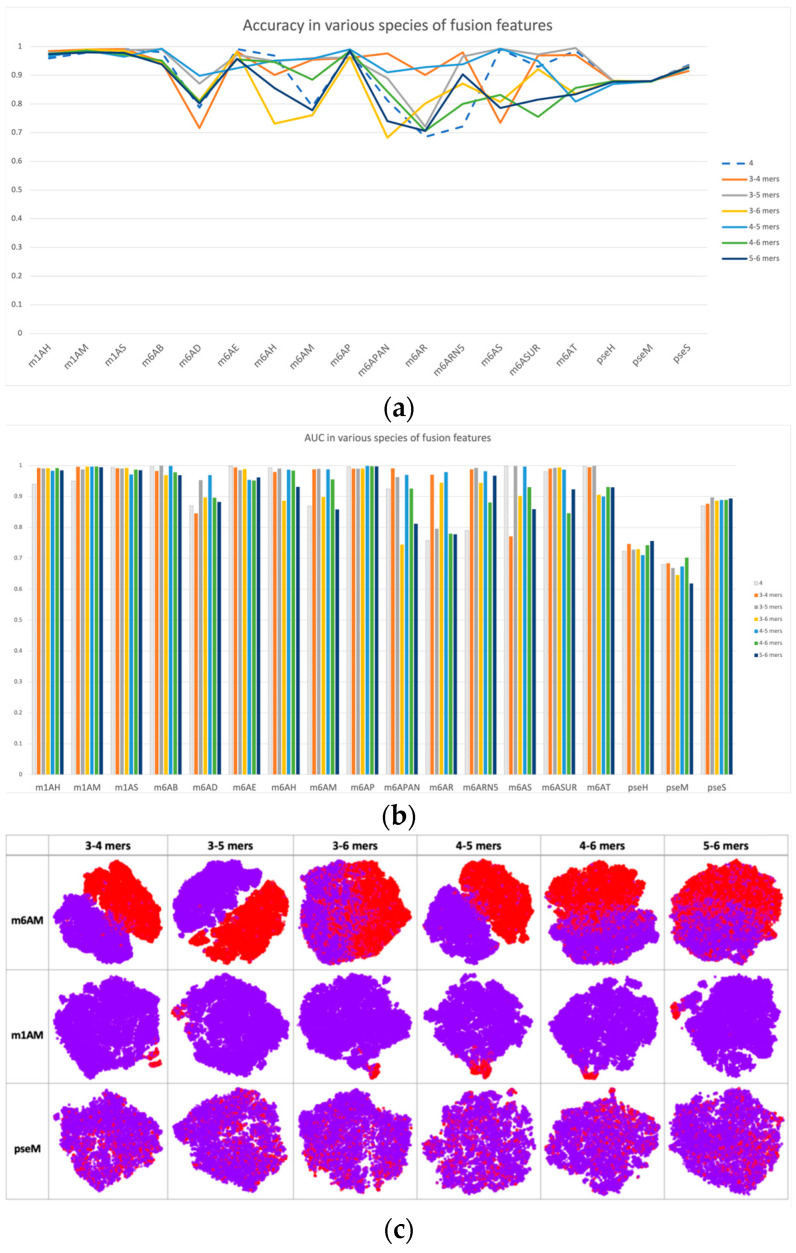
Performance and feature space distribution (with TSNE visualization) of fusion features in various datasets and modifications. (**a**) shows the criterion accuracy of fusion features and the 4-mer baseline on various modification, and criterion AUC is indicated on the bar chart of (**b**) with fusion features with 4-mer baseline. In the feature space distribution visualization (**c**), negative (in purple color) and positive (in red color) represent non-modified and true modified samples, respectively. The table-formed results of fusion features have been included in the supplementary file, Table S4.

Based on single-scale sequential patterns, we integrated different scales of k-mers as input patterns; for instance, the combination of 3-mer and 4-mer, and the combination of 5-mer and 6-mer. Figure 3 illustrates the results of this integration, where 4-mer was used as the baseline for comparison. In Figure 3a,b, we compared the performance of multiscale features with 4-mer features as our baseline. The performance improvements of multiscale features may seem insignificant at first, but they are consistent across multiple datasets. This consistency highlights how multiscale features complement each other, improving the model’s discrimination between positive and negative samples beyond what single-scale features alone can achieve. This was also confirmed by the feature space distribution in Figure 3c, where the clusters of positive and negative samples tended to be more separated when compared with single-scale cases, such as in datasets m1H and pseH. In these cases, the clusters of positive (red) and negative (purple) samples were easier to separate than in single-scale cases.

**Figure 4:**
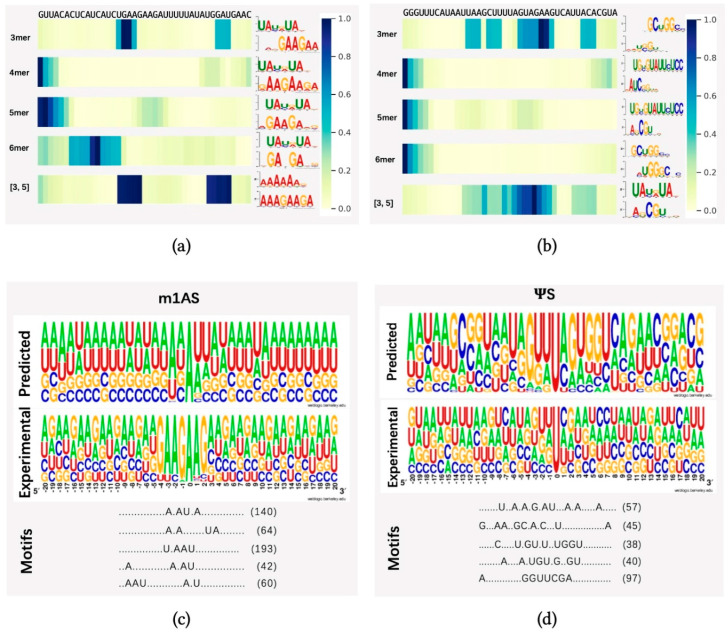
Visualization of attention score. In (**a**,**b**), we randomly selected one sequence from datasets m1AS and pseS, which represent modifications m1A and Ψ from dataset sacCer3, respectively (m1AS or pseS for short). Different choices of single k-values were shown in the heatmap, which were from the attention scores corresponding to the nucleotide composition (for instance: 3 mer means k = 3 in kmers, and [3, 5] stands for the fusion feature of 3 mer and 5 mer features) at each position of the input sequence. The right panel of subfigure (**a**,**b**) shows the motif patterns learned by our proposed method from different k-mer models. The corresponding motif patterns were visualized by the online motif comparison tool “TOMTOM”, with the above motifs from released RNA sequences of the species *Saccharomyces cerevisiae* in the database “CISBP-RNA single specie RNA”, while below are the motif patterns learned by our proposed method. (**c**,**d**) panels indicate the numbers of successive motif patterns found using the tool MotifX, the experimentally verified motifs generated from a positive dataset, and the motif patterns learned by our model from (**c**) m1AS and (**d**) pseS datasets, respectively. In each subfigure, the experimental panel shows the pattern visualization of positive sequences generated by TwoSampleLogo; the predicted panel shows the visualization of predicted substrates learned by our proposed model; and the motif patterns and their corresponding matched numbers are generated by MotifX, and the predicted top five substrates and their matched numbers are listed in the motifs panel.

To demonstrate whether any sequential region is of higher importance for methylation prediction, we randomly selected one sequence from dataset m1AS (m1A-modified sacCer3 dataset) and pseS (Ψ-modified sacCer3 dataset) and applied the attention mechanism to identify key regions from these sequences. We applied heatmap visualization of the corresponding normalized attention scores from the attention layer, using datasets m1AS and pseS as instances, as indicated in Figure 4a,b, respectively. Our model identified different regions under different sequential scales (3-mer, 4-mer, 5-mer, 6-mer) and a fusion of 3 and 5-mers. The top one motif patterns with the highest frequency learned by each model were also attached to the right side of each heatmap. The motif patterns were generated by an online motif comparison tool called “TOMTOM” [41,42]. The below pattern stands for our learned motif, and the above one was obtained by aligning the motif learned from the attention mechanism to a certain verified and released dataset (in this case, the database “CISBP-RNA single specie RNA” with species *Saccharomyces cerevisiae* was used for the alignment comparison). We found that the regions with higher attention scores differed among the models with multiple choices of k values, while in the fusion feature case, there might be more than one significant region of high importance, as there are two dark regions shown (i.e., m1AS, the fusion of three and five features). Compared with the experimentally verified motifs, our model can find important regions around the modified sites efficiently, as there are alignments of high similarity found in each case. In dataset m1AS, the similarity tended to be higher than in dataset pseS, which may be a reason for achieving higher performance in m1AS compared to pseS. Additionally, in the fusion feature case, there may be more than one significant region of high importance (i.e., m1AS, the fusion of three and five features).

To further analyze the differences between patterns obtained from our dataset and the verified one, we further employed the “MotifX” method [43]. This R-package tool is designed to extract overrepresented patterns from sequence datasets and was originally used for peptide sequence analysis. We used it to predict the successive substrate that frequently occurred near the modified sites in each individual dataset [31]. As shown in Figure 4c,d, the “predicted” panel indicates the motif patterns learned by our proposed model by inputting the training dataset and extracting the attention coefficients at each position. The “Motif” panel indicates the matched numbers of motif patterns found using the tool MotifX compared with our predicted result, and the “Experimental” panel indicates the experimentally verified motifs generated from a positive dataset and motif generation by the online tool “Weblogo” [44] from datasets m1AS and pseS, respectively. The predicted top five substrates and their matched numbers are listed in the motifs panel.

We discovered some successive motifs that occurred quite often, indicating that similar patterns tended to be important in different modifications and significantly contributed to certain types of modification. For example, comparing the subfigure Figure 4a,c, the ‘U.AAU’ pattern could be found very frequently, while in the dataset pseS, the pattern ‘C.GU.’ is frequently found. By comparing the visualization result from the attention score and MotifX (i.e., Figure 4a–d), we concluded that our proposed method could learn and obtain key regions of different modifications effectively. The motifs found in multi-scale features were highly similar to experimentally verified sequences, suggesting the existence of “biological grammars” that play a significant role in the procedure of RNA modification. We also observed that in methylation modifications, such as m1A, the “biological grammars” might be even more strict than in pseudo-uridine modification, as the patterns found in the m1A dataset are more obvious compared with the pseS dataset.

### 2.2. Comparative Analysis of Model Performance across Species and Methylations

In our study, we delved deeper into the performance of our model in cross-species scenarios, where we trained the model on one species and tested it independently on another. Given the limited availability of samples for each species, we chose three representative datasets for each modification for the cross-species evaluation, namely, sacCer3 (*Saccharomyces cerevisiae S288C*), mm10 (*Mus musculus*), and hg19 (*Homo sapiens*).

**Figure 5:**
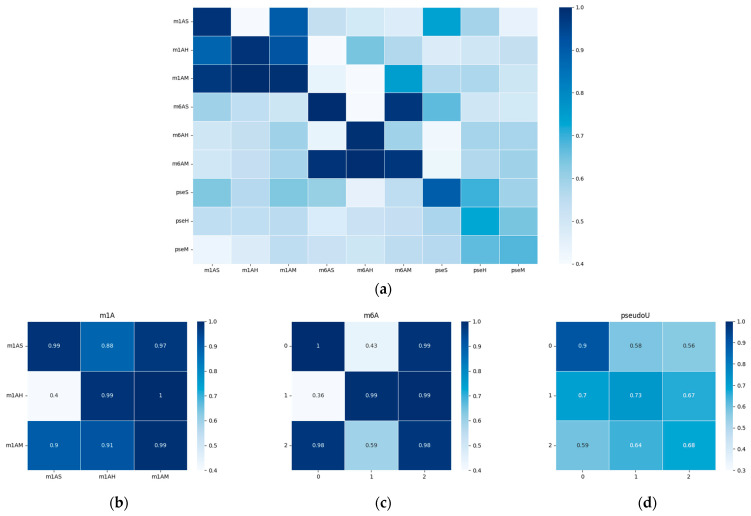
Heatmap of the performance of the cross-species validation; the *x*-axis represents the training datasets, while the *y*-axis represents the testing datasets. AUC values were chosen for visualization. The white color represents a lower AUC value, while blue indicates higher values. (**a**) Heatmap of the performances on all datasets with different modifications. (**b**–**d**) show the performance within each modification type, which corresponds to m1A, m6A, and Ψ modifications, respectively. A detailed AUC value was outlined in the supplementary file, Table S5.

To visualize the performance of our model, we generated a heatmap of the area under the receiver operating characteristic curve (AUC) and presented it in Figure 5. The results indicate that the model’s performance was significantly better when tested on species within the same evolutionary taxonomy than when tested on species from different evolutionary taxonomies. For example, the model trained on m6AH showed outstanding performance when tested on the m6AM dataset, as both modifications are found in mammals. When we focused on the diagonal metrices, which we have magnified in Figure 5b–d, we observed that different taxonomies showed different performance trends for m1A, m6A, and pseudo-uridine modifications. This finding suggests that methylation conservation at the sequential level is positively correlated within each modification type and across evolutionary taxonomies. Specifically, the model trained on hg19 showed relatively higher performance on the mm10 dataset, as both species belong to mammals. However, the model trained on the sacCer3 dataset, a fungal species, did not perform as well on the other two mammalian datasets for each single modification. It is worth noting that while the model shows robust performance within closely related species, there is a notable drop in accuracy when applied to more distantly related species. This highlights the challenges in building a universally applicable model for RNA modification prediction.

Furthermore, we observed that cross-species validation within a certain modification type yielded better results than when compared among different modifications. This observation was consistent with the hypothesis that there may be a “biological grammar” within each type of modification, and the “grammar” within methylations (i.e., m1A and m6A) tends to be stronger than within pseudo-uridine modification. As a result, the overall performance of the model tended to be higher for m1A and m6A modifications, and the differences between positive and negative results were much more pronounced compared to pseudo-uridine datasets, as shown in Figure 2c and Figure 3c.

### 2.3. Proposed Method Outperforms State-of-the-Art Methods

We have chosen some representative and newly accessible frameworks, namely the SRAMP [45] and WHISTLE [30], to compare with our proposed method, BERT-RNA. The deep learning framework DeepPromise [21], which is with only CNN, and DeepOME [46], which is with CNN incorporated with BLSTM, were also used in our comparison. The datasets for these chosen baseline methods were maintained as original species data, with the detailed comparison shown in Table 2.

Our results indicate that, in most cases, BERT-RNA outperformed conventional ML methods. When compared to methods with similar network structures, such as DeepPromise and MultiRM, in m6A modification, BERT-RNA exhibited a more outstanding performance, especially with larger datasets. Additionally, BERT-RNA demonstrated competitively balanced performance in pseudo-uridine modification prediction, maintaining relatively smaller biases in true-positive and true-negative predictions, as evidenced by the minimal differences in criteria “SE” and “SP”. While our model demonstrates balanced performance across multiple evaluation criteria, it is important to note that it may not outperform other tools in all metrics, as evident from Table 2.

## 3. Discussion

In this study, we proposed BERT-RNA, an approach to the identification of RNA modifications through the application of biological language learning that is based on sequential information. The predictive performance of BERT-RNA was investigated, and it demonstrated commendable performance across 18 benchmark datasets. These datasets cover modifications in three types (m6A, m1A, and pseU) across various species, highlighting the model’s superior and robust performance compared to contemporary sequence-based approaches.

Using DNABERT [48], a powerful natural language learning model pre-trained on large-scale genomic sequence data, was a strategic decision. This foundation enabled BERT-RNA to capture a wider range of sequential semantics from background genomes. We employed a multi-scale sequence processing strategy, drawing inspiration from word segmentation practices in natural language processing and the structure of previous pre-trained models. The RNA sequences were segmented into different scales (3 to 6 mers) of sequential patterns, creating ‘biological words’. This technique allowed for a detailed representation of RNA sequences and enabled the model to interpret the information learned from these varied scales of ‘biological words’ through the attention mechanism.

Despite the promising results, we acknowledge certain limitations and potential biases in our study. Further validation is required to determine the model’s applicability to untested RNA modifications and species. The performance of BERT-RNA, like many deep learning models, is significantly influenced by the quality and diversity of the training data. This dependence highlights the potential for biases in model predictions, emphasizing the need for careful dataset curation and model training. The need for model optimization and the development of more efficient computational frameworks is highlighted by the computational intensity of our approach, which presents another challenge.

The adaptation of DNABERT from DNA to RNA sequence analysis demonstrates the versatility and potential of pre-trained models in genomics. This cross-application highlights the critical role of evolutionary conservation and biological mechanisms underlying RNA modifications in interpreting the model’s performance. By analyzing the model’s ability to capture specific methylation patterns, validated against traditional motif-finding tools like TOMTOM, we highlight BERT-RNA’s capacity to elucidate the specificity of different methylation patterns. The learned motifs from BERT-RNA are highly consistent with known biological motifs, affirming its predictive accuracy and offering new avenues for the discovery of rare methylation patterns. However, further experimental verification is required to confirm the validity of these potential discoveries.

BERT-RNA provides a powerful extension to the analysis of RNA modifications, bridging the power of deep learning with the intricate requirements of genomic research. This work provides valuable insights into RNA modifications and their biological significance while also paving the way for future research to expand the application of deep learning models in genomics.

## 4. Materials and Methods

### 4.1. Datasets from Various Species

In this study, a diverse range of datasets were utilized for training and independent testing. To ensure comprehensive coverage, the datasets included samples from different modification types and species. Specifically, three types of modifications were considered, namely N1-methyladenosine (m1A), N6-methyladenosine (m6A), and pseudo-uridine (pseU), which are of larger data scale, making them more suitable for deep learning network analysis. Positive samples were collected from published work [31] and the RMBase2.0 database [49], containing at least one modification site on each sample sequence. Negative datasets were also included, consisting of sequences without any type of modification. As the datasets are from released work, a step of CDHIT for removing homologous sequences has already been taken in the preprocessing step. To avoid bias in the prediction result caused by the imbalance of positive and negative sample numbers, random under-sampling was employed during the training phase in some species with a positive-to-negative ratio larger than one to ten. Detailed performances caused by various positive and negative ratio numbers have been listed in the supplementary file, Table S1. In the testing sets, the ratio of positive to negative was kept imbalanced [50,51]. The detailed distribution of final datasets for each individual species has been listed in Table 3. To simplify the notation, the combination of each modification name and the first character of species name (or first three characters when duplicated name appeared) was used to denote the corresponding dataset with a certain modification. For instance, “m1AH” stands for m1A on the *Homo sapiens* dataset, “pseM” stands for pseudouridine modification on the *Mus musculus* dataset, “m6AP” stands for m6A modification on the *P. aeruginosa* dataset, while “m6APAN” stands for m6A modification on the *Pan troglodytes* dataset. Detailed dataset names and corresponding species names can be found in the supplementary file, Table S2.

### 4.2. Multi-Scale Information Processing Module

In this study, RNA sequences are separated into smaller segments called k-mers for detailed analysis. The term ‘k’ represents the length of these segments, which can be 3, 4, 5, or 6 nucleotides long, enabling examination of the sequences from multiple perspectives. This method transforms a single sequence into a series of overlapping segments, each providing a snapshot of the sequence’s structure. For example, the RNA sequence ‘GAUUACAU’ can be divided into overlapping segments such as ‘GAUUAC’, ‘AUUACA’, and ‘UUACAU’ when k is set to 6. This method produces a complete set of segments or ‘tokens’, comprising 4^k^ combinations for the k-mer lengths and five additional types ([CLS], [PAD], [UNK], [SEP], [MASK]) for specific processing requirements, such as indicating the beginning or end of a sequence [52]. Our model assigns a score to each token based on its importance, determined by the BERT encoder’s attention mechanism. This step is crucial for understanding which parts of the RNA sequence are most significant for predicting modifications, allowing for more interpretable results.

### 4.3. BERT Encoder Module

BERT (Bidirectional Encoder Representations from Transformers), introduced by Devlin et al. [53], revolutionized natural language processing (NLP) by being the first model to understand language contexts in both directions (left-to-right and right-to-left). DNABERT [48,54], a specialized version of BERT, designed to analyze genetic sequences, was adapted for our study due to its effectiveness in various NLP tasks and its deep understanding of language nuances. This model mirrors BERT’s structure, with 12 layers and specialized mechanisms for handling genetic data. It allows for the interpretation of RNA sequences by converting them to a DNA-like format, changing ‘U’ nucleotides to ‘T’. This conversion facilitates the use of DNABERT for RNA analysis, leveraging its ability to grasp complex patterns within genetic sequences. The pre-training of the model adapts to the specific characteristics of RNA, such as the length of the sequence, to ensure accurate predictions. DNABERT utilizes techniques such as the masked language model, a feature inherited from the original BERT model [52], to improve its learning from RNA sequences.

### 4.4. Fusion Feature Module

In this study, RNA sequences are analyzed at two different levels of detail, referred to as ‘scales’, to capture a broad range of information. The results are obtained from two sets of data: one from the first level of detail (*h_kmer_*_1_) and another from the second level (*h_kmer_*_2_). To combine these insights into a single, comprehensive analysis, a special technique called a ‘fusion gate’ (*F*) is used.

The fusion process operates by assessing the significance of data from each scale using a mathematical function called the sigmoid function. This function determines the amount of attention to allocate to each set of results based on their relevance. The formula we use is:

(1) $$F=sigmoid\left(W_{1}*\;h_{kmer1}+W_{2}*\;h_{kmer2}\right)$$

where *W*_1_ and *W*_2_ are adjustable factors that the model learns to optimize during training. This calculation gives us a combined score (*F*) that effectively merges the insights from both scales of analysis.

Finally, we calculate the overall analysis result (*h_M_*) by blending the two sets of results according to the combined score:

(2) $$h_{M}=F*\;h_{kmer1}+\left(1-F\right)*\;h_{kmer2}$$

This method ensures that our final analysis, represented by *h_M_*, benefits from the detailed insights gathered at both levels of detail.

### 4.5. Classification Module

Adversarial training is a technique used to improve the reliability and robustness of a model against intentional alterations in the data [55]. This is particularly important for the BERT model, which has a large number of parameters, as adversarial training helps prevent the model from overfitting the training data [56]. Specifically, we utilized a version of the Fast Gradient Method (FGM) [57,58] to classify texts, or in our case, RNA sequences.

The cross-entropy loss function (*L_CE_*) is the primary measure of model performance during training. It compares predicted and actual results to guide learning. In adversarial training, the function is adjusted to account for additional perturbations. This ensures that the model learns from challenging scenarios. The process works as follows: for each RNA sequence, we modify its representation slightly, guided by a calculation that identifies the smallest change that is likely to confuse the model. This is represented by ‘*r_adv_*’, a vector that quantifies the adjustment made to the representation of the sequence. The objective is to identify the worst-case changes that still result in accurate predictions by the model, thereby enhancing its robustness.

In mathematical terms, the original loss function (*L_CE_*) is adjusted by incorporating the effect of perturbations (*r_adv_*):

(3) LCEp,y|x+radv,θ=−ylogp−p−1−ylog1−pradv=Lr ,|r|≤ξarg minCEp,y|x,θ^ 

where *r* is a perturbation on the input, and $\hat{\theta }$ is a constant set to the current parameters of the model. The objective is to challenge the model with these slightly altered inputs and teach it to predict correctly despite them. This process involves calculating the gradient of the loss function with respect to the sequence representation (*s*), represented by ‘*g*’, to determine the direction of change that would most affect the model’s prediction. The gradient is scaled to a small value (*ξ*) and applied as a perturbation:

(4) $$r_{adv}=-\xi \frac{g}{||g|{|}_{2}}$$

where $g\;=\;\nabla_{s}\;L_{CE}\;\left(p,\;y|s,\;\theta \right)$. The performance of the model is then measured against these adversarially perturbed inputs to ensure accuracy, and the robustness of the model is summarized by an adversarial loss function:

(5) $$L_{adv}\left(\theta \right)=-\frac{\;1}{N}\sum_{n=1}^{N}L_{CE}(p_{n},\;y|S_{n}+r_{adv},n\},\;\theta ),$$

as proposed by Jin et al. [52].

### 4.6. Evaluation Metrics

Five-fold cross-validation and independent testing were used to evaluate the performance of the proposed model [59]. Four criteria: accuracy (Acc), sensitivity (Sn), specificity (Sp), and Mathew’s correlation coefficient (MCC) are used to evaluate the performance and defined as [60]:

(6) $$Sn=\frac{TP}{TP+FN}$$

(7) $$Sp=\frac{TN}{TN+FP}$$

(8) $$Acc=\frac{TP+TN}{TP+FP+TN+FN}$$

(9) $$MCC=\frac{TP\times TN-FP\times FN}{\sqrt{\left(TP+FP\right)\times \left(TN+FN\right)\times \left(TP+FN\right)\times \left(TN+FP\right)}}$$

where *TP* denotes positive samples that are correctly predicted as true modification sites, *TN* denotes negative samples that are correctly predicted to not contain any modification sites, *FN* is the number of true modification sequences that are falsely predicted as false modification sequences, and *FP* is the number of false modification sequences that are falsely predicted as true modification sequences.

In addition to these standard evaluation metrics, the receiver operating characteristic (ROC) curve was generated by plotting sensitivity against 1-specificity. This curve is another important indicator of the performance of the proposed method [61]. The area under the ROC curve (AUC value) ranges from 0 to 1, with a value of 1 indicating a perfect classification model. Therefore, the closer the AUC value is to 1, the better the prediction performance of the model and the higher the accuracy.

## 5. Conclusions

In this research, we proposed a multi-scale biological language-based deep learning model. Our model has demonstrated better performance than existing state-of-the-art methods across a wide range of RNA modifications and species. BERT-RNA uses innovative language-based deep learning techniques to decipher the complex sequential and functional semantics in genomic data. This not only improves prediction accuracy but also enhances our understanding of the biological mechanisms behind RNA modifications. The inclusion of an interpretable analysis mechanism in our model has been crucial in establishing a connection between identifying important sequential determinants and comprehensively analyzing their biological implications. In the future, validating the model’s ability to identify discriminative sequential patterns across different RNA modifications will require exploring larger datasets and a broader range of species. Investigating the potential for RNA sequence-based encoding schemes to elucidate the relationship between RNA modifications and peptide post-translational modifications is a promising avenue for future research. This leverages the insights provided by our study.

## Figures and Tables

**Table 2:** Comparison with state-of-the-art predictors for methylations. Here, Dataset Scale refers to the mentioned total sample number in each publication, consisting of training and independent testing sets. Performance from each predictor is from the corresponding publication. The optimal performance has been highlighted on bold.

Modification Type	Tool Name	Algorithm	Dataset Scale	Encoding Scheme	SE	SP	AUC	MCC
m1A	RAMPred [17]	SVM	6366 (*H. sapiens*) 1064 (*M. musculus*) and 483 (*S. cerevisiae*)	NCP, ANF	0.92	0.01	0.51	0.02
	iRNA-3typeA [18]	SVM	6366 (*H. sapiens*) and 1064 (*M. musculus*)	NCP, ANF	**0.98**	0.03	0.5	0.02
	DeepPromise [21]	CNN	5233	ENAC, one-hot and RNA word embedding	0.87	0.9	0.94	0.59
	MultiRM [24]	RNA embedding + LSTM + attention	16,380	RNA word embedding	0.64	0.8	0.78	0.45
	BERT-RNA (Ours)	Pretrained BERT model	4846	RNA word embedding	0.95	**0.98**	**0.99**	**0.79**
m6A	SRAMP [45]	RF	55,706 (full transcript mode) and 46,992 (mature mRNA mode)	one-hot, KNN score spectrum	0.44	0.9	0.29	0.79
	DeepPromise [21]	CNN	44,901, 11,656	ENAC, one-hot and RNA word embedding	0.39	0.9	0.25	0.76
	DeepOME [46]	CNN+BiLSTM	3052	One-hot	**0.97**	**1**	0.93	**0.99**
	CapNetwork [31]	CNN+BiLSTM+CapsuleNetwork	207,010	RNA word embedding	0.12	0.95	0.13	0.61
	Adaptive-m6A [31]	CNN+BiLSTM+attention	207,010	RNA word embedding	0.87	0.73	0.6	0.88
	MultiRM [24]	RNA embedding + LSTM + attention	65,178	RNA word embedding	0.82	0.78	0.86	0.6
	BERT-RNA (Ours)	Pretrained BERT model	422,994	RNA word embedding	**0.97**	0.98	**0.99**	0.94
	Porpoise [47]	emsenble learning framework	2472	BE, pseKNC, NCP, PSTNPss	0.82	0.75	0.74	0.59
Ψ	PSI-MOUSE [38]	SVM, RF, GLM, NB, DT	944	NCP, ND and Genome-derived features	0.86	**0.97**	**0.95**	**0.91**
	MultiRM [24]	RNA embedding + LSTM + attention	3137	RNA word embedding	**0.92**	0.76	0.85	0.69
	BERT-RNA (Ours)	Pretrained BERT model	8871	RNA word embedding	0.7	0.65	0.74	0.23

**Table 3:** Statistics of datasets in this research.

			N1-Methyladenosine (m1A)				N6-Methyladenosine (m6A)				Pseudouridine (pseU, Ψ)			
			Training Set		Testing Set		Training Set		Testing Set		Training Set		Testing Set	
Group	Species	Assembly	Positive	Negative	Positive	Negative	Positive	Negative	Positive	Negative	Positive	Negative	Positive	Negative
Bacteria	*P. aeruginosa*	ASM676v1	-	-	-	-	4082	34,559	1732	14,829	-	-	-	-
Mammal	*Pan troglodytes* (Chimpanzee)	panTro4	-	-	-	-	26,925	34,500	11,438	14,888	-	-	-	-
Mammal	*Macaca mulatta* (Rhesus)	rheMac8	-	-	-	-	27,264	34,493	11,573	14,895	-	-	-	-
Mammal	*Rattus norvegicus* (Rat)	rn5	-	-	-	-	35,102	34,466	14,894	14,922	-	-	-	-
Mammal	*Sus scrofa domesticus* (Pig)	susScr3	-	-	-	-	35,034	34,520	14,942	14,868	-	-	-	-
Plant	*Arabidopsis thaliana* (Thale cress)	TAIR10	-	-	-	-	14,328	34,475	6003	14,913	-	-	-	-
Mammal	*Mus musculus* (House mouse)	mm10	740	34,568	312	14,820	35,095	34,475	14,904	14,913	2322	2322	998	7261
Fungi	*Saccharomyces cerevisiae S288C*	sacCer3	858	34,567	362	14,281	47,367	34,574	20,304	14,814	1466	1466	650	7248
Mammal	*Homo sapiens* (Human)	hg19	1815	34,558	759	14,803	35,090	34,474	14,903	14,914	2405	2405	1030	7263
Insect	*Drosophila melanogaster* (Fruit fly)	BDGP6	-	-	-	-	4791	34,553	2028	14,835	-	-	-	-
Vertebrate	*Danio rerio* (Zebrafish)	danRer10	-	-	-	-	30,158	34,528	12,864	14,859	-	-	-	-

## Data Availability

All the data and codes involved in this research can be accessed at https://github.com/Moretta1/BERT-RNA on 27 February 2024.

## Supplementary Materials

The following supporting information can be downloaded at: https://www.mdpi.com/article/10.3390/ijms25052869/s1.
